# The effect of temperature on specific dynamic action of juvenile fall-run Chinook salmon, *Oncorhynchus tshawytscha*

**DOI:** 10.1093/conphys/coac067

**Published:** 2022-10-21

**Authors:** Vanessa K Lo, Benjamin T Martin, Eric M Danner, Dennis E Cocherell, Joseph J Cech, Jr, Nann A Fangue

**Affiliations:** Department of Wildlife, Fish and Conservation Biology, University of California Davis, Davis, 95616 CA, USA; Department of Theoretical and Computational Ecology, University of Amsterdam, 1098 XH Amsterdam, The Netherlands; NOAA Southwest Fisheries Science Center, Santa Cruz, 95060 CA, USA; Department of Wildlife, Fish and Conservation Biology, University of California Davis, Davis, 95616 CA, USA; Department of Wildlife, Fish and Conservation Biology, University of California Davis, Davis, 95616 CA, USA; Department of Wildlife, Fish and Conservation Biology, University of California Davis, Davis, 95616 CA, USA

**Keywords:** Aerobic scope, cost of digestion, fish, metabolism

## Abstract

Juvenile fall-run Chinook salmon (*Oncorhynchus tshawytscha*) in the Sacramento–San Joaquin River Basin experience temporally and spatially heterogenous temperature regimes, between cool upper tributaries and the warm channelized Delta, during freshwater rearing and outmigration. Limited water resources necessitate human management of dam releases, allowing temperature modifications. The objective of this study was to examine the effect of temperature on specific dynamic action (SDA), or the metabolic cost associated with feeding and digestion, which is thought to represent a substantial portion of fish energy budgets. Measuring SDA with respect to absolute aerobic scope (AAS), estimated by the difference between maximum metabolic rate (MMR) and standard metabolic rate (SMR), provides a snapshot of its respective energy allocation. Fish were acclimated to 16°C, raised or lowered to each acute temperature (13°C, 16°C, 19°C, 22°C or 24°C), then fed a meal of commercial pellets weighing 2% of their wet mass. We detected a significant positive effect of temperature on SMR and MMR, but not on AAS. As expected, there was no significant effect of temperature on the total O_2_ cost of digestion, but unlike other studies, we did not see a significant difference in duration, peak metabolic rate standardized to SMR, time to peak, percent of meal energy utilized, nor the ratio of peak O_2_ consumption to SMR. Peak O_2_ consumption represented 10.4–14.5% of AAS leaving a large amount of aerobic capacity available for other activities, and meal energy utilized for digestion ranged from 5.7% to 7.2%, leaving substantial remaining energy to potentially assimilate for growth. Our juvenile fall-run Chinook salmon exhibited thermal stability in their SDA response, which may play a role in maintaining homeostasis of digestive capability in a highly heterogeneous thermal environment where rapid growth is important for successful competition with conspecifics and for avoiding predation.

## Introduction

Chinook salmon (*Oncorhynchus tshawytscha*) are an anadromous fish species native to the North Pacific Ocean that have faced major population reductions under increasing anthropogenic stress (Yoshiyama *et al.,* 1998; Noakes *et al.,* 2000). The Sacramento–San Joaquin River Basin (SSJRB) of California supports some of the most heavily impacted populations of Chinook salmon, where there are four seasonal runs: fall, late-fall, winter and spring—the latter two of which are endangered and threatened, respectively (Moyle *et al.,* 2017). Within the SSJRB, which encompasses the southernmost range for Chinook salmon, extensive engineering projects have altered flow and temperature regimes, degraded habitats and eliminated access to historical spawning areas (Yoshiyama *et al.,* 1998). Chinook salmon are predicted to be further impacted by climate change as decreases in reservoir storage will reduce river flows and increase water temperatures (Moyle *et al.,* 2017). For ectotherms such as fish, temperature is a critical variable that affects virtually all aspects of an organism’s physiology and biochemistry (Huey and Kingsolver, 1993; Hochachka and Somero, 2002). Paradoxically, the fate of Chinook salmon in the SSJRB depends on human management because water temperatures are now artificially regulated by dam releases (Yates *et al.,* 2008).

Temperature-dependent bioenergetic processes such as metabolism (*Ṁ*O_2_) are commonly measured in fishes as a proxy for physiological performance (Fry, 1971). One such metabolic performance metric is absolute aerobic scope (AAS)—the difference between the minimum and maximum metabolic rate (MMR) as measured by oxygen consumption rate (Farrell, 2016). The minimum metabolic rate, termed standard metabolic rate (SMR), represents a fish’s basic need for oxygen, while MMR is a fish’s capacity to deliver additional oxygen to support activities beyond this basic need (Chabot *et al.,* 2016b). Thus, AAS provides an estimate of the surplus energy available to an organism that can be invested into fitness-related functions (e.g. growth, digestion, locomotion, avoiding predation, reproduction, etc.), providing a snapshot of a fish’s energy budget under specific measurement conditions (Clark *et al.,* 2013). Because this energy surplus is finite, fish must make tradeoffs among various functions such as growth, development and digestion (Sokolova *et al.,* 2012). Understanding how energy balance changes with respect to temperature may be helpful in predicting tolerance limits, population success and response to future climate impacts for Chinook salmon in California and other fishes (Pörtner, 2010; Poletto *et al.,* 2017; Steell *et al.,* 2019; Jutfelt *et al.,* 2021; Zillig *et al.,* 2021).

**Figure 1:**
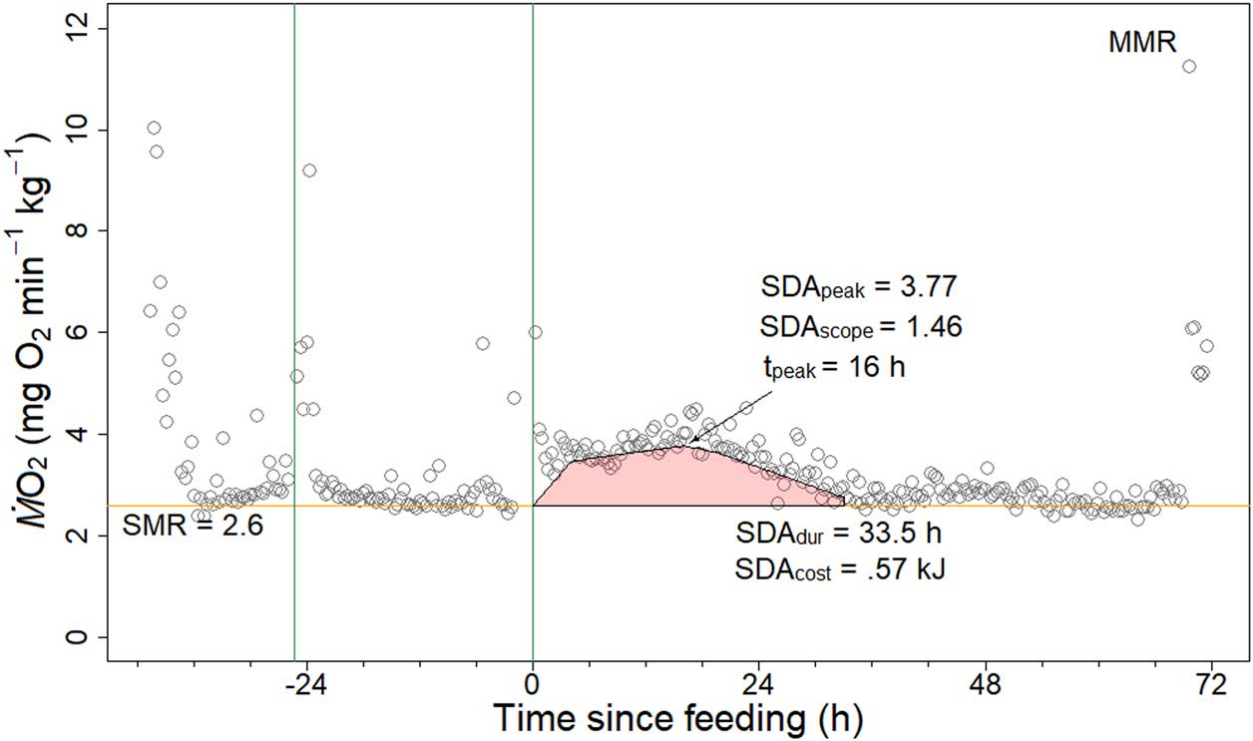
A representative continuous recording of the *Ṁ*O_2_ (mg O_2_ min^−1^ kg^−1^) from a juvenile Chinook salmon (25.9 g, 12.9 cm fork length) acclimated to 16°C and tested at 16°C using a respirometry system at UC Davis. At the first vertical bar (Time: −24), the fish was removed, sham-fed and returned to the vessel. At the second vertical bar (Time: 0), the fish was removed and force-fed 2% of its wet body weight with formulated pellets and returned to the vessel for up to 72 h. A quantile regression is used to estimate the SMR from pre-feeding *Ṁ*O_2_, indicated by the horizontal line, whereas SDA is estimated from post-feeding *Ṁ*O_2_. SDA is considered terminated when the regression converged with the SMR + 5%. The duration of the SDA response is 33.5 h and is noted as SDA_dur_. SDA_cost_ is estimated by integrating the area between the curve and SMR (marked polygon area) and is reported in kJ by assuming 1 g of O_2_ is associated with the release of 13.6 kJ of energy (Cho *et al.,* 1982). SDA_scope_ (mg O_2_ min^−1^ kg^−1^) and t_peak_ (h) are indicated by the arrow. MMR was collected at the end of the experiment by chasing the fish in a bucket to exhaustion and then returning it immediately to the respirometry chamber.

Feeding and digestion are thought to represent a substantial portion of fish energy budgets, as the resulting increase in *Ṁ*O_2_ can last for hours or days (Soofiani and Hawkins, 1982). The metabolic cost of feeding is referred to most commonly in the literature as specific dynamic action (SDA) and is defined as the increase in metabolism associated with ‘ingestion, digestion, absorption and assimilation of a meal’ (Kleiber, 1975; Secor, 2009). A fish’s SDA is measured by continuously recording metabolic rate after feeding, providing a complete profile of the postprandial metabolic response (Fig. 1; Table 1). SDA is known to be affected by many factors such as meal size, meal composition, feeding frequency, hypoxia and body temperature (Jobling, 1981, 1983; Fu *et al.,* 2005; Eliason *et al.,* 2007; Eliason and Farrell, 2014; Tirsgaard *et al.,* 2015; Steell *et al.,* 2019). Body temperature is a primary determinant of the shape and dyamics of the SDA response. Warmer temperatures increase peak *Ṁ*O_2_ during digestion (SDA_peak_), increase peak *Ṁ*O_2_ standardized to baseline (Peak_net_), shorten the duration of the postprandial response (SDA_dur_) and reduce the time to peak (t_peak_), effectively temporally compressing the SDA response (Jobling, 1981; McCue, 2006; Eliason *et al.,* 2011; Sandblom *et al.,* 2014). In contrast, warming temperatures have negligible effects on the total cost (SDA_cost_) and ratio of SDA_cost_ to meal energy content (SDA_coef_) of the postprandial response (McCue, 2006). Importantly, the temporal compression of SDA with increasing temperatures is expected to reduce the proportion of AAS remaining during SDA_peak_—termed postprandial residual aerobic scope (PRAS)—and may have major implications for fitness as tradeoffs must be made between using AAS for SDA or locomotion, growth and other processes (LeGrow and Beamish, 1986; Sandblom *et al.,* 2014; Jutfelt *et al.,* 2021).

**Table 1:** Definition of variables used to quantify the postprandial metabolic response to feeding

Variable	Definition
SMR (mg O_2_ min^−1^ kg^−1^)	Baseline metabolic rate of postabsorptive individuals
SDA_cost_ (kJ)	Total O_2_ cost of the postprandial response, calculated as the area under the *Ṁ*O_2_ curve bounded by SMR and converted to kJ using an oxycalorific coefficient (Cho *et al.,* 1982)
SDA_peak_ (mg O_2_ min^−1^ kg^−1^)	Postprandial peak in *Ṁ*O_2_ (mg O_2_ min^−1^ kg^−1^)
Peak_net_ (mg O_2_ min^−1^ kg^−1^)	SDA_peak_ minus SMR
t_peak_ (h)	Time from feeding to SDA_peak_ in hours
SDA_dur_ (h)	Duration of time from feeding to when *Ṁ*O_2_ is no longer significantly greater than baseline (SMR + 5%) (Chabot *et al.,* 2016a)
SDA_scope_	Ratio of SDA_peak_ to SMR
SDA_coef_ (%)	SDA_cost_ divided by the digestible energy content of the meal

While studies investigating the effect of temperature on SDA in fishes are not uncommon, there are only a handful investigating the effect in the *Oncorhynchus* genus (Thorarensen and Farrell, 2006; Eliason *et al.,* 2007, 2008; Eliason and Farrell, 2014), and none in juvenile Chinook salmon. Additionally, assessing the effect of temperature on SDA, AAS and PRAS together has been described for very few species (Pang *et al.,* 2010, 2011; Sandblom *et al.,* 2014). In particular, the effect of temperature on SDA is important to understand for juvenile Chinook in the SSJRB due to the diversity of runs and life histories in this watershed. Despite increasingly severe and frequent drought conditions that will raise water temperatures and strain reservoir capacities, a rigid temperature criteria of 13.3°C (7-day average of daily maximums, 7-DADM) for endangered winter-run Chinook embryo rearing during summer forces a wider temperature range than is natural for the dam-truncated watershed (USFWS, 1999; Zillig *et al.,* 2021). The lower Sacramento River mainstem regularly experiences temperatures exceeding 20°C by late spring, and although juvenile Chinook can tolerate short-term exposures to sublethal temperatures (25°C+), the duration of exposure is expected to increase in the future (Myrick and Cech, 2004). As increasing water temperatures are expected to reduce PRAS, understanding how temperature affects SDA, AAS and PRAS is necessary to contextualize the portion of AAS dedicated to feeding and digestion and to define the role of feeding in juvenile Chinook energy budgets (Norin and Clark, 2017). The aim of this study was to measure the effects of temperature on SDA variables and AAS in juvenile fall-run Chinook salmon across a range of ecologically relevant temperatures experienced in the SSJRB.

## Materials and Methods

### Experimental animals

Juvenile fall-run Chinook salmon were transported from Coleman National Fish Hatchery (Anderson, CA, USA) via an aerated transport tank that maintained oxygen levels of >90% of air saturation. All fish were from the same cohort and experienced the same rearing conditions, and the number of families was unknown. Fish (*n* = 200) were transferred to the Center for Aquatic Biology and Aquaculture (University of California, Davis, CA, USA) on 25 June 2017 and were reared in two (590 l) tanks with air-equilibrated well-water flow-through (3 l min^−1^). Well-water salinity was <0.5 practical salinity unit and temperature was kept at 16°C under natural photoperiod conditions for Davis, CA, USA (38.5 N, 121.7 W), for at least 3 weeks prior to experimentation. Fish were fed 3 mm commercial pellet feed (50% protein, 12% oil, 9% moisture, 3% fibre, 12% ash, 14.6 kJ/g digestible energy; Skretting, Toole, UT, USA) ad libitum ration over a 12-h period daily. Experimental fish were size selected from the source tank because SDA responses can only be directly compared between conspecifics of a certain age/size that are consuming identical meals (McCue, 2006). Mean body mass (*P* = 0.070) and total length (*P* = 0.098) did not differ significantly among the five temperature treatments (Table 2). All experimental protocols and fish care methods were approved by the UC Davis Institutional Animal Care and Use Committee, protocol #18196.

**Table 2:** Temperature effects on postprandial metabolism in juvenile Chinook salmon

Variable	Temperature				
	13 °C	16 °C	19°C	22 °C	24 °C
*n*	12	10	9	12	11
SMR (mg O_2_ min^−1^ kg^−1^)	2.0 ± 0.06^a^	2.4 ± 0.10^b^	2.7 ± 0.14^b^	3.3 ± 0.16^c^	4.2 ± 0.05^d^
MMR (mg O_2_ min^−1^ kg^−1^)	9.3 ± 0.53^a^	10.7 ± 0.33^ab^	10.3 ± 0.59^ab^	11.2 ± 0.42^b^	11.5 ± 0.54^b^
AS (mg O_2_ min^−1^ kg^−1^)	7.3 ± 0.52	8.3 ± 0.38	7.5 ± 0.52	7.9 ± 0.43	7.2 ± 0.55
Wet mass (g)	28.1 ± 0.68	26.3 ± 1.26	29.8 ± 1.43	26.4 ± 0.60	28.9 ± 0.99
Fork length (cm)	13.4 ± 0.11	13.0 ± 0.18	13.6 ± 0.19	13.2 ± 0.12	13.5 ± 0.13
Total length (cm)	14.4 ± 0.12	14.0 ± 0.16	14.5 ± 0.21	14.2 ± 0.12	14.5 ± 0.14
SDA_cost_ (kJ)	0.40 ± 0.04	0.51 ± 0.09	0.49 ± 0.07	0.47 ± 0.04	0.50 ± 0.05
SDA_peak_ (mg O_2_ min^−1^ kg^−1^)	2.7 ± 0.11^a^	3.4 ± 0.14^b^	3.6 ± 0.19^b^	4.3 ± 0.18^c^	5.3 ± 0.11^d^
Peak_net_	0.73 ± 0.06	0.94 ± 0.09	0.88 ± 0.14	1.00 ± 0.08	1.02 ± 0.11
t_peak_ (h)	19.3 ± 1.8	13.2 ± 2.2	15.4 ± 1.7	15.0 ± 1.6	14.0 ± 1.3
SDA_dur_ (h)	43.4 ± 2.7	39.0 ± 4.1	39.5 ± 3.0	38.2 ± 2.8	36.6 ± 3.4
SDA_scope_	1.36 ± 0.03^ab^	1.39 ± 0.04^a^	1.33 ± 0.06^ab^	1.31 ± 0.03^ab^	1.24 ± 0.03^b^
SDA_coeff_ (%)	5.7 ± 0.5	7.2 ± 1.1	6.4 ± 0.9	6.5 ± 0.5	6.4 ± 0.7
PRAS (mg O_2_ min^−1^ kg^−1^)	6.5 ± 0.5	7.3 ± 0.3	6.7 ± 0.5	6.9 ± 0.5	6.2 ± 0.5
Remaining scope for activity (%)	89.6 ± 0.9	88.6 ± 1.0	88.2 ± 1.9	86.6 ± 1.6	85.5 ± 1.5

Values are reported as means ± s.e. Different superscript letters represent significantly different values between acute temperature treatments (*P* < 0.05; Tukey’s test).

### Respirometry

*Ṁ*O_2_ (mg O_2_ min^−1^ kg^−1^) of individual fish was measured using intermittent flow respirometry using a seven-chamber system fabricated at UC Davis. Each 1.5-l acrylic respirometry chamber was mounted in a 284-l aerated and UV-sterilized water bath surrounded by black curtains to minimize disturbance. The intermittent flow cycle was set such that each flush period was 5 min, the wait period was 1 min and the recirculating closed period was 7–10 min depending on temperature, during which the oxygen content of the water was recorded every second using a fibre-optic oxygen dipping probe (Loligo Systems, Viborg, Denmark) inserted into the respirometer through a water-tight rubber stopper. Oxygen levels within the respirometry chamber were not allowed to decline to <80% saturation at the end of each measurement period to ensure the fish did not become hypoxic and stressed (Svendsen *et al.,* 2016). Each respirometer had a DC recirculation pump to maintain water mixing during the measurement period and to minimize flow disturbances to the fish. Flush and recirculation periods were controlled using Autoresp™ software (Loligo Systems, Viborg, Denmark). *Ṁ*O_2_ values were calculated from the linearly declining O_2_ content of the water inside the respirometer during each closed period, and limited to slopes with an R^2^ > 0.96 (Svendsen *et al.,* 2016). Prior to each experiment, oxygen probes were calibrated with oxygen-free distilled water and fully aerated distilled water. Oxygen-free distilled water was created by adding 1 g sodium sulphite (Na_2_SO_3_; Spectrum Chemical Manufacturing Corp., CA, USA) to 100 ml of distilled water, while fully aerated distilled water was created by bubbling ambient air into 100 ml of water for 20 min. Both calibration measurements were conducted inside the experimental water bath to reduce temperature fluctuations.

Following a 3-week acclimation to 16°C, individual fish were tested at one of five acute temperatures: 13°C, 16°C, 19°C, 22°C or 24°C. Due to natural diel fluctuations in the facility’s well water source, water temperatures had a fluctuation of up to **±**1.0°C. The experimental protocol was identical for each acute temperature. In total, 9–12 fish per acute temperature were included (Table 2). Fish were fasted for 24 h in individual holding tanks before being placed randomly in a respirometry chamber at the acclimation temperature of 16°C. Figure 1 presents a representative trace of *Ṁ*O_2_ data over the course of an SDA experiment for an individual fish.

After a 1-h adjustment period to the respirometer, temperature in the water bath was either held at 16°C or changed at 2°C/h to 13°C, 19°C, 22°C or 24°C. Upon reaching the acute temperature, *Ṁ*O_2_ measurements began, to provide data for SMR estimates. Because attempts to coerce the fish to feed voluntarily in the respirometer were unsuccessful, a force-feeding protocol was used to administer the meal (personal communication from Dr Erika J. Eliason, University of California, Santa Barbara). The next morning (−24 h in Fig. 1), each fish underwent a sham-feeding procedure (completely identical to force-feeding but without food ingestion) to habituate the fish to the process of force-feeding and to assess the handling effect on *Ṁ*O_2_ (Eliason *et al.,* 2007, 2008). After an additional 24 h (0 h in Fig. 1), the fish was again removed and force-fed a meal using 3 mm pellets consistent in caloric content, composition and digestible energy content (McCue, 2006). Target meal sizes were 2% of wet body mass because pilot experiments showed that larger rations often resulted in partial or total regurgitation. Additionally, mean meal sizes of 2.18% and 1.16% were measured for wild and hatchery juvenile Chinook salmon, respectively, from the Nisqually River delta, Puget Sound, Washington, justifying our target meal size (Davis *et al.*, 2018). Ultimately, mean meal sizes were 1.81% ± 0.03 for all fish and did not differ significantly among temperature treatments (*P* = 0.15). The force-feeding protocol consisted of lightly anaesthetizing fish with a buffered solution of tricaine methanesulfonate (0.03 g/l; MS-222; Syndel, Ferndale, WA, USA) until loss of equilibrium, followed by measurement of wet mass and manual administration of a meal with rubber-tipped forceps (Eliason *et al.,* 2008). Fish were then returned into their respirometers and postprandial *Ṁ*O_2_ was measured for 72 h (0–72 h in Fig. 1). Any pellets regurgitated were syphoned out, counted and multiplied by the known mean mass of a dry pellet, to eliminate bias introduced by hydrated pellets (Eliason *et al.,* 2007). One quarter of fish regurgitated pellets within the respirometer, and was typically limited to one or two pellets, equivalent to 0.02–0.04 g, or 4–8% of the intended meal size. Regurgitation did not trend with temperature. At the end of the 72-h period, fish were removed and manually chased to exhaustion with a hand net until they no longer responded to contact of the net with their caudal fin (usually between 3 and 6 min), then returned to respirometry chambers immediately for an MMR measurement (Cutts *et al.,* 2002; Svendsen *et al.,* 2012). At the end of the experiment, fish were euthanized in a lethal buffered tricaine methanesulfonate (0.5 g/l) solution, then measured to the nearest 0.01 g and 1.0 mm.

Background microbial *Ṁ*O_2_ in each respirometer chamber was measured at three time points in each experiment: during the sham feeding procedure, during the feeding procedure and post-experiment. *Ṁ*O_2_ values for individual fish were corrected by grouping background *Ṁ*O_2_ values by acute temperature, fitting an exponential model to each dataset, then subtracting the predicted values from each fish’s *Ṁ*O_2_ trace (Svendsen *et al.,* 2016).

### Data and statistical analysis

*Ṁ*O_2_ was recorded using Autoresp™ software (Loligo Systems, Viborg, Denmark) and data analyses were performed using R studio (version 3.6.1; http://R-project.org/). *Ṁ*O_2_ values included in analysis were required to have an R^2^ > 0.96, resulting in an average loss of 7.5% of total *Ṁ*O_2_ values collected. For SMR estimates, *Ṁ*O_2_ values were filtered to remove the hours representing handling stress as indicated by the sham-feeding protocol, and the 48 h after feeding (time zero, Fig. 1) to remove the elevated values of the SDA response period. SMR and all variables of SDA were calculated using the *fishMO2* package and R script provided by Chabot *et al.* (2016a). This script also included the R package *quantreg* (Koenker, 2011), which fit nonparametric quantile regressions to the data to estimate SMR and SDA, where values of tau (τ), the penalty parameter (λ) and the tolerance value were set at 0.2%, 12% and 5%, respectively, based on recommendations given by Chabot *et al.* (2016a). For estimating the SDA curve, a non-parametric quantile approach was used, which allows some percentage of the observations, set by τ, to fall below the estimated line (Chabot *et al.,* 2016a, 2016b). Chabot *et al.* (2016b) recommend choosing the value of τ based on the optimal method used to estimate SMR. In this study, the recommended method for calculating SMR was non-parametric quantile regression in 43 fish and mean of the lowest normal distribution in 11 fish. This justified using the same value of 0.2 for both non-parametric quantile regression calculations of SMR and τ for SDA calculations. Setting τ = 0.2 allowed 20% of the *Ṁ*O_2_ values to fall below the estimated SMR and SDA lines. λ was set to 12, as it is recommended to be larger than the duration of an activity cycle, which for most fish is one per day lasting half a day or less (Chabot *et al.,* 2016a). The tolerance value of 5% terminated the SDA curve when the quantile fit reached SMR + 5% (Fig. 1; Chabot *et al.,* 2016a). The effect of sham feeding on *Ṁ*O_2_ was assessed by inspecting data for each individual fish. Because sham feeding typically elevated *Ṁ*O_2_ and subsided after 4 h, a 4-h period was removed before fitting the model and SDA was assumed to follow a straight line joining the origin of the SDA response (time zero, Fig. 1) to the first value predicted by the fitted line, as recommended by Chabot *et al.* (2016a). SDA variables were calculated from these fitted curves to describe the post-feeding *Ṁ*O_2_ metrics (Table 1) following Secor (2009). SDA_cost_ was converted to kJ from the area bounded by SDA and SMR (polygon in Fig. 1) by assuming that 1 g of oxygen is associated with the release of 13.6 kJ of energy (Cho *et al.,* 1982). In contrast, MMR was limited to one value per fish due to having one opportunity to elicit MMR.

**Figure 2:**
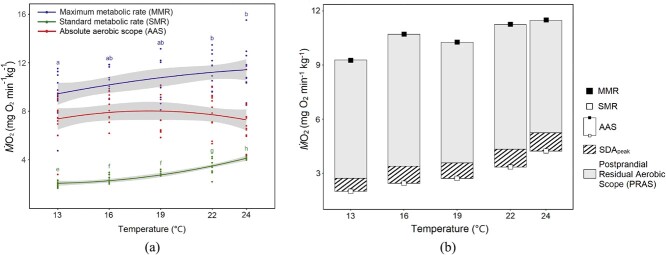
**(a)** The effect of temperature on SMR, MMR and AAS in juvenile Chinook salmon reared at 16°C and tested at 13°C, 16°C, 19°C, 22°C and 24°C. Solid blue dots and line represent MMR fit to a second-order polynomial described by MMR (mg O_2_ min^−1^ kg^−1^) = 4.550 + 0.479x − 0.008x^2^ where x is temperature in °C. Solid green dots and line represent SMR fit to a second-order polynomial described by SMR (mg O_2_ min^−1^ kg^−1^) = 4.791 – 0.443x + 0.018x^2^ where x is temperature in °C. Solid red dots and line represent AAS fit to a second order polynomial described by AAS (mg O_2_ min^−1^ kg^−1^) = − 0.241 + 0.921x − 0.026x^2^ where x is temperature in °C. **(b)** Relationships between SDA_peak_, SMR, MMR and AAS at 13°C, 16°C, 19°C, 22°C and 24°C. SMR and MMR are represented by the open and closed squares, respectively, and AAS is the difference between the two, represented by the grey rectangle. SDA_peak_ is represented by the hatched area.

All metabolic and SDA variables were grouped according to temperature treatment and examined for differences using a one-way ANOVA, with differences between groups tested using Tukey’s honest significant difference when relevant. Results were considered significant at *P* < 0.05. All values are reported as mean ± SE unless otherwise noted. Data analyses were completed using R (R Core Team, 2019). Correlations between water temperature and SMR, MMR and AAS were fitted with polynomial regression lines using lowest AIC model selection (Fig. 2a). An exception was MMR, where a linear regression model fit best. However, we have chosen to also report a polynomial regression line for MMR because it is more ecologically logical, and the difference in AIC between the linear and polynomial regression models was <0.5 AIC units.

## Results

Typically, mean *Ṁ*O_2_ showed an elevated and somewhat variable pattern following the sham and actual feedings (Fig. 1). The mean postprandial increase was of much greater mean duration than that following the sham feeding and peaked (t_peak_) at 19.3, 13.2, 15.4, 15.0 and 14.0 h at 13°C, 16°C, 19°C, 22°C and 24°C, respectively, though it was not significantly different among temperature treatments (*P* = 0.123; Table 2). The duration of the SDA response (SDA_dur_) trended negatively with temperature from 43.4 to 36.6 h, but was not significant among temperature treatments (*P* = 0.602). As expected, SDA_peak_ increased with temperature (*P* < 0.001) except between 16°C and 19°C (*P* = 0.908). However, when SMR was subtracted from peak values (Peak_net_), there were no significant differences among treatments (*P* = 0.183). The ratio of peak to SMR (SDA_scope_) decreased with temperature from 1.36 to 1.24 and was significantly different (*P* = 0.0411) between 16°C and 24°C (*P* = 0.046). The mean energetic cost of the SDA response (SDA_cost_) as well as the mean percentage of the ingested meal energy consumed by SDA_cost_ (SDA_coef_) were not significantly different (*P* = 0.666 and *P* = 0.743, respectively) (Table 2).

Mean SMR increased significantly (*P* < 0.001) with increasing temperature at each of the five tested temperatures except between 16°C and 19°C (*P* = 0.504), and was fitted to the equation SMR (mg O_2_ min^−1^ kg^−1^) = 4.791–0.443x + 0.018x^2^ where x is temperature in °C (Fig. 2a; Table 2). Mean MMR was significant (*P* = 0.015) between 13°C and 22°C (*P =* 0.035) and 13°C and 24°C (*P* = 0.016) and was fitted to the equation MMR (mg O_2_ min^−1^ kg^−1^) = 4.550 + 0.479x − 0.008x^2^ where x is temperature in °C (Fig. 2a; Table 2). Mean AAS ranged from 7.2 to 8.3 mg O_2_ min^−1^ kg^−1^ but was not significantly different (*P* = 0.54) and was fitted to the equation AAS (mg O_2_ min^−1^ kg^−1^) = −0.241 + 0.921x − 0.026x^2^ where x is temperature in °C (Fig. 2a; Table 2). In the context of AAS, the peak SDA response comprised 10.4–14.4% of AAS, leaving a mean PRAS of 6.2–7.3 mg O_2_ min^−1^ kg^−1^ or 85.5–89.6% of AAS, although neither was significantly different among treatments (*P* = 0.244 and *P* = 0.57, respectively) (Fig. 2a, b; Table 2).

## Discussion

### SDA variables

The SDA responses from our salmon were remarkably similar across all temperature treatments, with SDA_cost_, Peak_net_, t_peak_, SDA_dur_, SDA_coef_ and SDA_scope_ all not significantly different. SDA_peak_ was significantly different across temperatures, but this significance disappeared once standardized to individual fish’s SMR (Peak_net_) and corresponded to postprandial increases of 124–136% of SMR. Therefore, we conclude that the SDA response in this population of juvenile fall-run Chinook salmon shows a large degree of thermal independence. This level of thermal insensitivity was unexpected, given the influence ambient temperature exerts on the biologic rates of ectotherms. Our temperatures represented an ecologically relevant range experienced by juvenile fall-run Chinook salmon in upper tributary rearing grounds (10–14°C) and in the Sacramento–San Joaquin Delta (~25°C) as they outmigrate, suggesting that juvenile fall-run Chinook have substantial plasticity in their digestive response (Columbia Basin Research, University of Washington, 2022).

While not significant, our fish did exhibit a slight trend towards increased SDA_peak_ and decreased SDA_dur_ of the metabolic response with increasing temperature. Effects of temperature on SDA_peak_ and SDA_dur_ are documented in many ectotherm species, but inconsistent in magnitude across species or even within the same family, as evidenced by the differing thermal susceptibility of these metrics in three cyprinid species (Pang *et al.,* 2010). Variability in SDA response of fishes is also explained by lifestyle (i.e. active swimmers vs. sit-and-wait ambush predators) due to differences in the capacity of the central cardio-respiratory, digestive and locomotor systems (Pang *et al.,* 2011; Jutfelt *et al.,* 2021). Steell *et al.* (2019) observed higher metabolic rates during meal digestion rather than from exhaustive exercise in tropical lionfish, with SDA_peak_ exceeding active metabolic rate by as much as 1.7 times. Even small meals occupied 64% of AAS in lionfish at 26°C, whereas our salmons’ peak occupied only 10.4–14.4% of AAS at all temperatures.

One can also express SDA_peak_ as a percentage of MMR, or the oxygen-transporting capacity of the cardiovascular system. For our fish, SDA_peak_ corresponded to 30.5–46.6% of MMR, leaving a substantial amount of aerobic capacity available. Studies in juvenile rainbow trout, where SDA was compared to MMR rather than AAS, have suggested higher percentages of MMR consumption at SDA_peak_. LeGrow and Beamish (1986) estimated SDA_peak_ for 10–15 g rainbow trout fed 2% of their body mass with diets of varying protein and lipid contents at 15°C, to be between 60% and 80% of MMR. However, MMR was calculated using an equation given by Rao (1968) and MMR can vary considerably between individuals. Similarly, for 10–20 g juvenile rainbow trout (*Oncorhynchus mykiss*) at 15°C, Alsop and Wood (1997) measured satiation-fed (~3% of body mass on average) metabolic rate as ~70% of MMR, although fish were fed as a group within a tank prior to individual transfer to respirometers so exact meal size was unknown. Eliason *et al.* (2007) estimated average SDA_peak_ in adult rainbow trout (503.4 ± 10.7 g, mean ± SEM) to consume 53% (minimum) and 69% (average) of MMR, using a MMR given by Kiceniuk and Jones (1977).

Generally, it appears both juvenile and adult salmonids can utilize a large proportion of their aerobic scope for digestion if needed, though our study suggests juvenile Chinook in the SSJRB require less aerobic capacity to digest a meal. Definitive conclusions on trends between juvenile and adult fish require further study due to differences in methods, diet composition and species. Additionally, it is possible that the dynamics of SDA changes with age due to dietary protein needs and the role of protein synthesis in the postprandial increase in metabolic rate (Seth *et al.,* 2009). In well-studied rainbow trout aquaculture, the optimal dietary protein level for optimal growth decreases from 50% to 35% from very young trout to adult maintenance diet (Hilton and Slinger, 1981). For adult rainbow trout, Eliason *et al.* (2007) found that isoenergetic diets with varying protein and lipid levels, which significantly alter protein utilization and deposition, had no effect on SMR, SDA_peak_, t_peak_ or SDA_cost_. The substantial aerobic capacity remaining in our juvenile Chinook salmon at SDA_peak_ at all temperatures suggests that digestion is an important function and may be attributed to their need to grow rapidly at this life stage. Measurements of *Ṁ*O_2_ in fed and fasted Chinook salmon and rainbow trout (*O. mykiss*) forced to swim at critical swimming speeds found that MMR remained the same, but that critical swimming speed was lower in the fed fish (Alsop and Wood, 1997; Thorarensen and Farrell, 2006). For these *Oncorhynchus spp.,* the metabolic processes associated with digestion and assimilation are prioritized, potentially at the expense of maximum sustained swimming performance.

Our salmons’ relatively small and constant SDA_coef_ of 5.7–7.2% indicates that a small proportion of ingested meal energy went toward the SDA response, leaving substantial absorbed energy remaining to be allocated to growth (LeGrow and Beamish, 1986). However, the pellet diet we provided is an energetically high-density food (14.6 kJ/g digestible energy), which likely led to lower SDA_coef_ values than would be found with fish consuming natural prey items. Davis *et al.* (2018) assessed gut contents of juvenile Chinook salmon from the Nisqually River delta in Puget Sound, Washington, and estimated energy density of stomach contents to be 5.32 ± 2.94 and 4.47 ± 2.62 kJ/g for wild and hatchery fish, respectively, and stomach fullness as a percent of fish wet weight to be 2.18 ± 3.58% and 1.16 ± 2.80%. Given our salmons’ meal size of 2% corresponding to a mean of 0.51 g and with a mean SDA_cost_ of 0.47 kJ, values of SDA_coef_ with more realistic meal energy densities would be 17.4% and 21.0% for wild and hatchery fish, respectively. Although Davis *et al.*’s (2018) data are from a different watershed, similar types of prey are consumed by juvenile fall-run Chinook salmon in the lower Mokelumne River and yolo bypass, both of which are located within the SRB (Merz, 2002; Goertler *et al.,* 2018). Additionally, juvenile salmon augment their foraging behaviour by preferentially consuming calorically valuable prey and consuming a greater quantity of prey when calorically valuable prey are not available (Goertler *et al.,* 2018). These estimated SDA_coef_ values are also within the range of 11.9–32.3% for Biwa trout (*Oncorhynchus rhodurus*) fed 1.0–3.3% of body weight with rainbow trout (*Salmo gairdneri*) or ayu (*Plecoglossus altivelis*) fillets (Miura *et al.,* 1976). Due to the variability of SDA_coef_ depending on meal energy density and size, we caution comparing SDA_coef_ values between studies without taking into account these details. Investigating the SDA response in juvenile Chinook using natural prey items is an avenue for further study.

Our salmons’ SDA_scope_ of 1.24–1.39 was lower than the 1.5–2.5 times SMR range reported for many different fish (Jobling, 1981; McCue, 2006; Secor, 2009), and SDA_dur_ was similar to that reported for the congeneric rainbow trout (*O. mykiss*) fed a meal of 2% body mass (Medland and Beamish, 1985; LeGrow and Beamish, 1986; Eliason *et al.,* 2007). Fish likely face a tradeoff between ingesting large, infrequent meals vs. smaller, more frequent meals due to the inverse relationship between SDA_dur_ and SDA_peak_. SDA_dur_ typically increases with increasing meal size and is variable depending on fish size and meal composition (Jobling, 1981). However, it is thought that SDA is dominated by relatively fixed metabolic costs created by the upregulation of digestive processes, so it is possible that regular feeding reduces the costs of constantly up- and down-regulating the digestive system (Boyce and Clarke, 1997). Fish fed multiple meals have mixed results, with juvenile cod (*Gadus morhua*) exhibiting a cumulative effect of increased *Ṁ*O_2_ with each meal and a maximum observed after the third or fourth meal (Soofiani and Hawkins, 1982). In contrast, lionfish had reduced costs when feeding frequently vs. feeding singularly (Steell *et al.,* 2019). For juvenile Chinook salmon, we suspect the dynamics of multiple feedings to more resemble that of juvenile cod due to greater similarities in size, prey choice and lifestyle. Because we measured a single instance of feeding using easily digestible pellets, our SDA values likely represent the lower end of postprandial energy consumption for juvenile Chinook in the wild.

### SMR, MMR, AAS and PRAS

Our salmons’ SMR, MMR and AAS (Fig. 2a; Table 2) were consistent with those from previous reports for the same species of a similar mass tested from 12°C to 26°C, although MMR was elicited using an incremental swimming protocol in contrast to the chase protocol in the present study (Poletto *et al.,* 2017). Juvenile Chinook had relatively constant aerobic capacities over the range of acute temperatures, which was maintained by matching the increase in SMR with an equivalent increase in MMR (Fig. 2a). Thermal insensitivity of AAS has been documented in another Californian *Oncorhynchus* species, with hatchery and wild *O. mykiss* tested on the Lower Tuolumne River showing an ability to maintain 95% of maximum AAS across a wide temperature range of 17.8–24.6°C (Verhille *et al.,* 2016). Previous studies on salmonids from more northern latitudes showed an AAS peak or plateau at high temperatures, which then plummets when critical temperatures are reached (Farrell, 2016). However, in the present study and for other *Oncorhynchus* species located in the Central Valley of California, clear peaks or plummets are lacking (Verhille *et al.,* 2016). We attempted to measure SDA at 25°C, but found that exposure to this temperature for longer than 24 h proved fatal. Mortality from chronic exposure to temperatures above 24°C is well documented in juvenile salmonids, although the underlying mechanism is not well understood (Myrick and Cech, 2002). Interestingly, maintaining a high level of swimming performance and aerobic capacity up to nearly lethal temperatures has been shown in multiple juvenile Chinook populations from a range of latitudes along the West coast of the USA, as well as in adult sockeye salmon (*Oncorhynchus nerka*) from the Fraser River, British Columbia, Canada (Eliason *et al.*, 2013; personal communication from Dr Zillig, University of California, Davis). Our attempts to test fish at 25°C may have been additionally hampered by a lack of ram ventilation due to static respirometers rather than swim tunnels.

By measuring both SDA variables and AAS in individual fish, we could assess PRAS, an ecologically relevant metric of available excess energy after consumption of a meal. Our salmon’s PRAS of 6.2–7.3 mg O_2_ min^−1^ kg^−1^ was equivalent to a remaining scope for activity of 85.5–89.6% of AAS—quite a large proportion. The remaining energy must fuel all other activities for a given fish and it is possible that additional stressors, strenuous activity or warmer, sublethal temperatures could reduce PRAS (Jutfelt *et al.,* 2021). However, it is suggested that juvenile salmonids modify their behaviour to maximize AAS via foraging in prey-dense mainstem habitats, followed by retreating to cooler thermal refugia such as tributaries (Brewitt *et al.,* 2017).

### Limitations and assumptions

One limitation of our study is that we did not investigate assimilation efficiency (AE)—the fraction of ingested food that is incorporated into biological tissue. AE is measured by calculating absorption minus defecation and excretion during an organism’s gut transit time and can be affected by food type, frequency of ingestion and temperature (Pouil *et al.,* 2018). In addition, the absorption of specific nutrients and elements can also vary with temperature. For example, Van Campenhout *et al.* (2007) showed that decreasing the temperature from 25°C to 15°C in common carp (*Cyprinus carpio*) caused no change in cadmium AE, but a significant decrease in zinc AE. Although an increase in temperature typically increases enzymatic activity and decreases gut transit time, resulting in no change in AE, some lizard species exhibit reduced AE at extreme temperatures (Plasman *et al.,* 2019). Thus, the PRAS maintained across our test temperatures may not be indicative of the use value of the meal provided, potentially affecting growth. Additionally, in the wild, digestion is affected by behavioural mediation such as movement to different temperatures, reduction of meal size, increased meal frequency and intentional regurgitation (Jutfelt *et al.,* 2021).

Our study was conducted with hatchery juvenile Chinook under temperature-controlled, well-oxygenated conditions, with optimal feed and without additional environmental stressors. In contrast, wild fish must obtain prey, escape predators, choose suitable habitat and cope with variable environmental conditions, creating much more complex dynamics when it comes to prioritizing energetic demands. Recently, there has been an improved understanding of how the introgression of hatchery- and wild-origin fish has reduced fitness (Araki *et al.,* 2008), eroded life history diversity (Carlson and Satterthwaite, 2011) and resulted in drastically increased hatchery contributions to spawning populations (Willmes *et al.,* 2018). However, little is known about the direct consequences on digestion, energetics or physiological response to temperature. Unfortunately for the fate of wild Chinook in the Central Valley, over 90% of fish captured in the ocean fishery in 1992 and 2002 for fall-run Chinook salmon were of hatchery origin (Barnett-Johnson *et al.,* 2007)—a consequence of over a half-century of large-scale hatchery propagation (Sturrock *et al.,* 2019). For our study, this makes our use of hatchery-origin fish more relevant than in other watersheds where wild fish retain a larger genetic difference from their hatchery counterparts, although it does not discount the importance of social and behavioural cues in energy use that may differ for the two settings.

Lastly, our study was conducted with one acclimation temperature of 16°C, with acute exposure to test temperatures. Acclimation temperature is known to affect thermal performance curves, with physiological responses occurring at time scales ranging from minutes to weeks (Schulte *et al.,* 2011). For juvenile fall-run Chinook sourced from Coleman hatchery, AAS in fish acclimated to 11°C, 16°C and 20°C and measured at acute temperatures from 8°C to 25°C had similarly shaped responses among acclimation temperatures, with more dispersion between acclimation temperatures occurring at the lower range of acute temperatures (Zillig *et al.*, in press, CJFAS). This suggests that across these acclimation and acute temperatures, the patterns observed in our SDA metrics may not vary dramatically with changes in acclimation temperatures, although this would need to be confirmed in future studies. For much longer term acclimations lasting days to weeks, metabolic thermal compensation may occur, where a sudden change to a new thermal condition alters metabolic metrics (such as SMR, MMR and AAS) but the fish is able to compensate to some degree over time (Sandblom *et al.,* 2014). Ultimately, neither acclimation nor acute temperature changes take into account the behaviour of wild fish and their decisions in thermal regulation.

## Conclusions

The results of our study suggest that moderate temperatures (13–24°C) seen throughout the SSJRB are not a critical factor when it comes to the cost of digestion in fall-run juvenile Chinook salmon. However, extended periods of sublethal temperatures (24°C+) are likely to increase in frequency and duration, lowering survival among juvenile Chinook. We believe prey availability is likely to be a more important factor, as evidence suggests that abundant prey resources may mitigate the negative effects of elevated temperature on fish growth (Brewitt *et al.,* 2017; Lusardi *et al.,* 2020). Additionally, physiological plasticity in the form of thermal acclimation is well documented for Chinook salmon. Palmisano *et al.* (2000) found that Chinook salmon increased heat-shock protein 90 expression in heart, muscle, brain and gill tissues after a 5-h exposure to 21.6°C, indicating an acute compensatory mechanism. Such rapid compensatory mechanisms and the importance of growth in juvenile Chinook salmon may explain the minimal effects of temperature on our SDA variables. In conclusion, juvenile Chinook salmon are exposed to both cool riverine temperatures in upper-watershed rearing grounds and to warmer temperatures within the estuaries and bay as they migrate to the ocean. The diversity of life history strategies among runs of Chinook salmon in the SSJRB result in juveniles rearing within the watershed nearly year round (Brandes and McLain, 2001). The thermal stability of their SDA responses may play a role in maintaining homeostasis in digestive capability in a highly heterogeneous environment, where rapid growth is important for successful competition with conspecifics and for avoiding predation (Sogard, 1997; Beamish and Mahnken, 2001).

## Supplementary Material

Web_Material_coac067

## Data Availability

The data needed to reproduce the statistical analyses and figures in this study are publicly archived on Figshare at https://doi.org/10.6084/m9.figshare.20422302.
