# Supplementary material for: The effect of temperature on specific dynamic action of juvenile fall-run Chinook salmon, Oncorhynchus tshawytscha
Source: Conserv Physiol. 2022 Oct 21;10(1):coac067. doi: 10.1093/conphys/coac067 (PMC9616469; doi:10.1093/conphys/coac067)

Supplementary Materials

Supplementary Table 1. Mean ± standard error of factorial aerobic scope (FAS) at each test temperature, calculated by the equation: FAS = MMR/SMR. FAS was examined for differences using a one-way ANOVA, with differences between groups tested using Tukey’s honest significant difference when relevant. Results were considered significant at *p* < 0.05.

| Temperature | Factorial Aerobic Scope (FAS) |
| --- | --- |
| 13 | 4.66 ± 0.266^a^ |
| 16 | 4.46 ± 0.260^ab^ |
| 19 | 3.81 ± 0.184^abc^ |
| 22 | 3.44 ± 0.178^cd^ |
| 24 | 2.72 ± 0.142^d^ |

Supplementary Figure 1: The effect of temperature on aerobic scope (AAS) and Peak_net_ in juvenile Chinook salmon reared at 16℃ and tested at 13, 16, 19, 22, and 24℃. Solid red dots and line represent AAS fit to a second order polynomial described by AAS (mg O_2_ min^-1^ kg^-1^) = – 0.241 + 0.921x – 0.026x^2^, where x is temperature in ℃. Peak_net_ is represented a linear regression described by Peak_net_ (mg O_2_ min^-1^ kg^-1^) = 0.446 + 0.025x, where x is temperature in ℃.


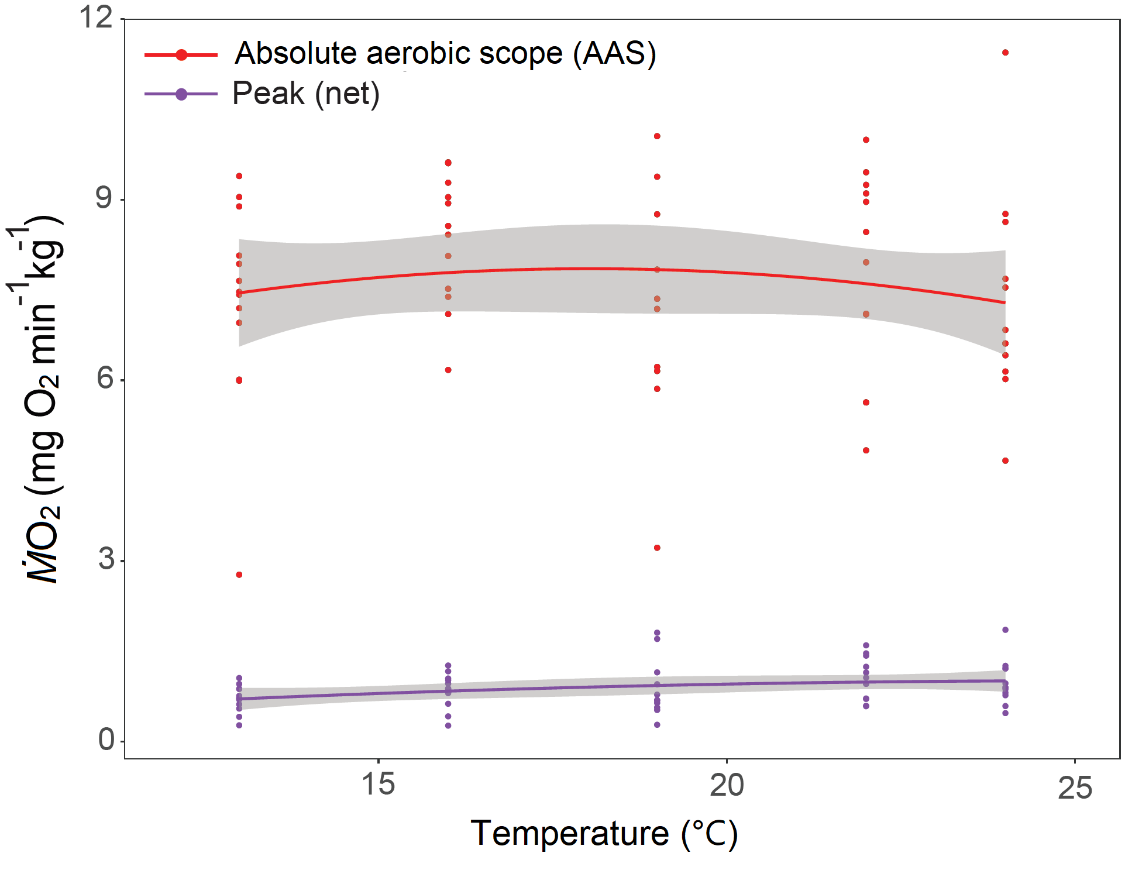


Supplementary Figure 2. The effect of temperature on (a) SDA_cost_, (b) SDA_peak_, (c) peak_net_, (d) t_peak_, (e) SDA_dur_, (f) SDA_scope_ and (g) SDA_coef_ in juvenile Fall-run Chinook salmon reared at 16℃ and tested at 13, 16, 19, 22, and 24℃. Differences were examined using a one-way ANOVA, with differences between groups tested using Tukey’s honest significant difference when relevant. Results were considered significant at *p* < 0.05.


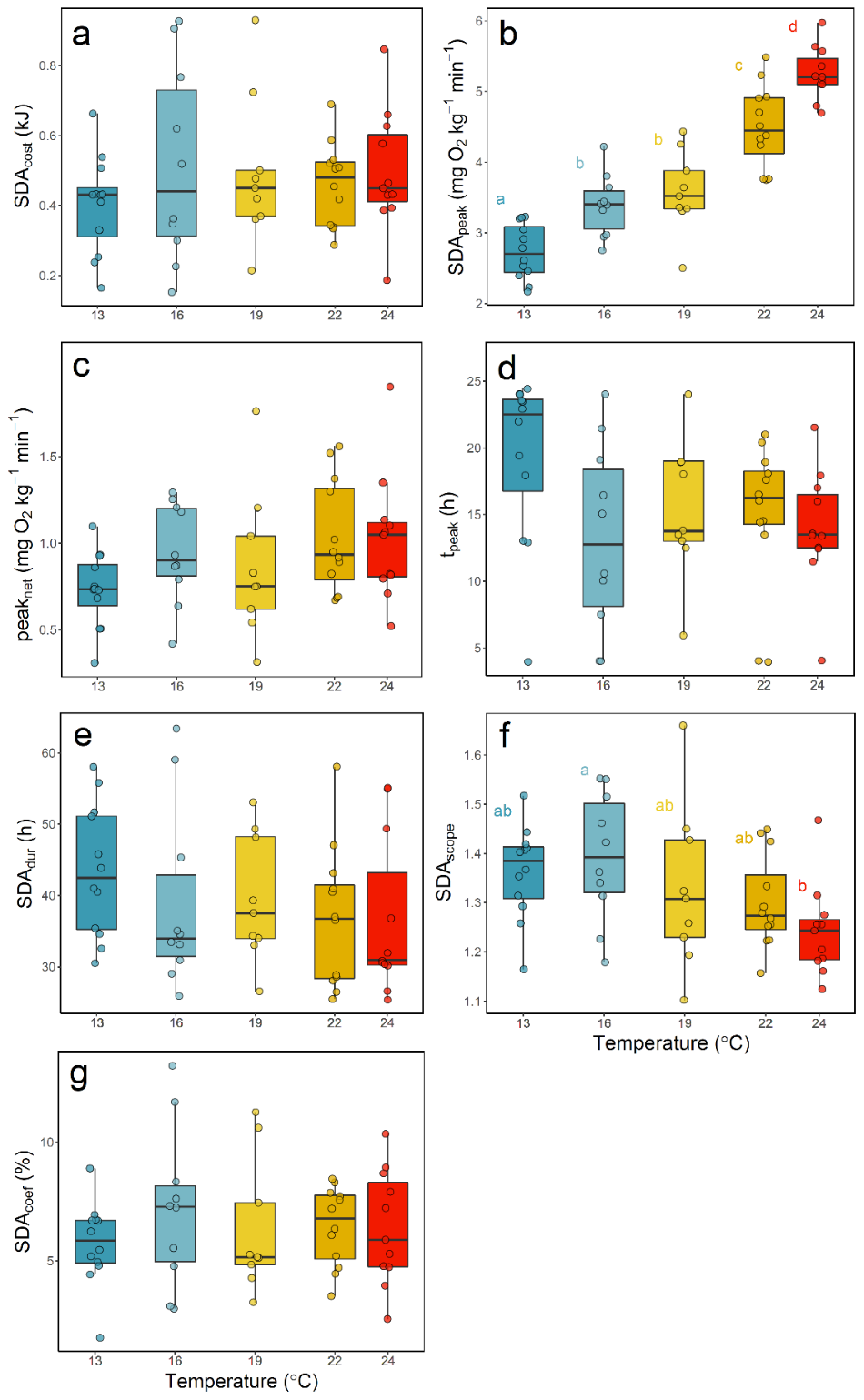

Supplement: Web_Material_coac067 [file web_material_coac067.zip › Supplementary Materials.docx]
